# Temperature Dependence of Nonlinear Elastic Moduli of Polystyrene

**DOI:** 10.3390/polym17081008

**Published:** 2025-04-08

**Authors:** Andrey V. Belashov, Anna A. Zhikhoreva, Yaroslav M. Beltukov, Irina V. Semenova

**Affiliations:** Ioffe Institute, Russian Academy of Sciences, 26, Polytekhnicheskaya, 194021 St. Petersburg, Russiayaroslav.beltukov@mail.ioffe.ru (Y.M.B.)

**Keywords:** polystyrene, elastic properties, nonlinear elastic moduli, temperature dependence

## Abstract

Nonlinear elastic properties of polymers and polymer-based composites are essential for accurate prediction of their response to dynamic loads, which is crucial in a wide range of applications. These properties can be affected by strain rate, temperature, and pressure. The temperature susceptibility of nonlinear elastic moduli of polymers remains poorly understood. We have recently observed a significant frequency dependence of the nonlinear elastic (Murnaghan) moduli of polystyrene. In this paper we expanded this analysis by the temperature dependence. The measurement methodology was based on the acousto-elastic effect, and involved analysis of the dependencies of velocities of longitudinal and shear single-frequency ultrasonic waves in the sample on the applied static pressure. Measurements were performed at different temperatures in the range of 25–65 °C and at different frequencies in the range of 0.7–3 MHz. The temperature susceptibility of the nonlinear moduli *l* and *m* was found to be two orders of magnitude larger than that of linear moduli λ and μ. At the same time, the observed variations of *n* modulus with temperature were low and within the measurement tolerance. The observed tendencies can be explained by the shift of nonlinear moduli towards higher frequencies with increasing temperature.

## 1. Introduction

Polymers are a class of materials characterized by long chains of repeating molecular units, which impart unique mechanical, thermal, and chemical properties. Their versatility and adaptability make them essential in a wide range of applications, from everyday consumer products to advanced industrial components [1,2]. The ability to tailor the properties of polymers through chemical modifications and processing techniques has led to their widespread use in various fields, including packaging, automotive, aerospace, electronics, and healthcare.

In recent years, the incorporation of nanomaterials into polymer matrices has given rise to polymer nanocomposites, which exhibit significantly enhanced properties compared to their conventional polymer counterparts [3,4,5,6]. By integrating nanoscale fillers, such as nanoparticles, nanotubes, or nanofibers, into the polymer matrix, one can achieve improvements in mechanical strength, thermal stability, barrier properties, and electrical conductivity. These enhancements are primarily attributed to the high surface area-to-volume ratio of nanomaterials, which facilitates better interaction with the polymer matrix and leads to a more effective load transfer at the nanoscale. Polymer nanocomposites enable the development of lightweight materials that can meet the demanding performance requirements of modern applications [7,8,9,10,11]. For instance, in the automotive and aerospace industries, reducing weight while maintaining structural integrity is crucial for improving fuel efficiency and overall performance. Additionally, the unique properties of polymer nanocomposites make them suitable for innovative applications in fields such as energy storage, environmental remediation, and biomedical devices.

Despite the numerous advantages offered by polymers and polymer nanocomposites, understanding their mechanical behavior under various loading conditions remains a critical area of research [2,12]. The mechanical properties of polymer materials are significantly affected by the strain rate, working temperature and pressure. The vast majority of the existing research was focused primarily on the *linear* elastic characteristics of these materials. The frequency and temperature dependence of the linear elastic moduli has been studied in various polymers and polymer nanocomposites [13,14,15,16,17,18,19].

The study of *nonlinear* elastic properties has become increasingly important in recent years, as it provides critical insights into the behavior of materials under various loading conditions. Nonlinear elasticity refers to the phenomenon where the stress-strain relationship deviates from linearity, particularly at higher stress levels [20]. This behavior is essential for an accurate prediction of the material response to dynamic loads, which is crucial in applications ranging from structural engineering to geophysics and biophysics [21,22,23,24,25,26]. Understanding the nonlinear elastic properties is particularly important for polymers and other soft materials, since they may experience large deformations under relatively small applied forces, necessitating a departure from classical linear elasticity models [27].

Several methods have been developed for experimental evaluation of nonlinear elastic properties of materials. These techniques include dynamic acousto-elasticity [28,29,30], second harmonic generation [31], Brillouin scattering [32], coda wave interferometry [33], strain solitary waves [34], as well as Lamb [35] and Rayleigh [36] waves. Nonlinear elastic properties of materials can be described by a set of nonlinear elastic moduli, which characterize the deviation of a stress-strain relationship from linear behavior. Different sets have been proposed by Landau and Lifshitz [37], Murnaghan [38], and Thurston and Brugger [39]. The nonlinear elastic moduli defined in these models are interrelated and can be derived from one another. In particular, Murnaghan’s model describes the nonlinear elastic behavior of isotropic solid materials using three third-order elastic moduli (denoted as *l*, *m*, and *n*) and two second-order Lamé moduli (λ and μ). Specifically, in Murnaghan’s model, the elastic energy density is expressed as follows [38]:

(1) $$\Pi =\frac{\lambda +2\mu }{2}I_{1}^{2}-2\mu I_{2}+\frac{l+2m}{3}I_{1}^{3}-2mI_{1}I_{2}+nI_{3},$$

where $I_{1}=\mathrm{tr}E$, $I_{2}=\left[{\left(\mathrm{tr}E\right)}^{2}-\mathrm{tr}\left(E^{2}\right)\right]/2$, and $I_{3}=\det E$ represent the invariants of the Green–Lagrange strain tensor E. This model is the most comprehensive for isotropic materials undergoing small but finite deformations, as Equation (1) incorporates all conceivable second- and third-order terms that remain invariant under any rotation of the coordinate system. In the context of stress-strain relationships, Lamé moduli describe linear dependence and are connected to other linear elasticity moduli, with μ being the shear modulus and $\lambda +\frac{2}{3}\mu$ representing the bulk modulus. Nonlinear Murnaghan moduli *l*, *m*, *n* describe the deviation from linear elasticity as indicated by Equation (1), with various combinations of *l*, *m*, *n* playing distinct roles in diverse nonlinear phenomena.

The nonlinear elastic moduli are valuable for predicting fatigue damage [40], understanding thermoelastic properties of crystalline solids [41], analyzing radiation damage [42], and studying phenomena such as creep and thermal aging [31]. Overall, nonlinear parameters have been found to be more responsive to structural changes in materials compared to their linear counterparts [43].

In general, the nonlinear moduli of polymers depend both on the frequency and the temperature, forming their complex nonlinear viscoelastic behavior [44]. There are a number of methods to study the nonlinear viscoelastic response of different polymeric materials. However, most of them are devoted to studying the polymer melts and solutions and studying the shear flow in these materials. To mention a few, there are techniques of large amplitude oscillatory shear (LAOS) [45], medium amplitude oscillatory shear (MAOS) [46] and medium amplitude parallel superposition (MAPS) [47]. However, the elastic energy expansion (1) is not applicable to fluids.

The nonlinear viscoelastic properties of solid amorphous polymers have also been studied in the literature [48]. Typically, research in this area focuses on low-frequency regimes. Standard methodologies, such as stress relaxation and creep tests, are commonly employed to evaluate the behavior of these materials [49]. At the same time, the aforementioned methods such as LAOS have also been applied to glassy polymers [50]. Another method is mechanical spectral hole burning (MHBS) [51,52]. The observed frequency-dependent nonlinear viscoelastic behavior can be explained by molecular relaxation processes, whose rates depend on temperature, stress, and strain [53,54]. However, the observed frequency and temperature dependence of nonlinear viscoelastic properties often reflects the specific experimental methodology and does not encompass the full set of nonlinear moduli. Consequently, the frequency and temperature dependencies of the Murnaghan moduli *l*, *m*, *n* demand further exploration. This investigation is particularly essential for the MHz frequency range, which encompasses ultrasonic non-destructive testing techniques. Understanding these dependencies is vital for predicting the mechanical properties of solid amorphous polymers in real-world applications and for establishing a comprehensive methodology for their systematic evaluation.

The significant temperature dependence of the nonlinear elastic moduli has been observed in vitreous silica [55], metal-matrix composites [56], and constrained steel blocks [57]. Nonlinear elastic moduli demonstrated significant changes at the phase transition temperature [58]. Exploiting the thermal susceptibility of the nonlinear properties of elastic waves has opened a new way to improve the accuracy, precision, and reliability of the use of acoustic nonlinearity in elastic wave imaging [59]. However, the temperature susceptibility of nonlinear elastic moduli *l*, *m*, *n* in polymers remains poorly understood.

Polystyrene, a commercially significant thermoplastic polymer, is known for its excellent mechanical properties, optical clarity, and chemical resistance. Structural elements fabricated from pure polystyrene and polystyrene-based composites operate at different environmental conditions and are subjected to various dynamic loads. The performance of these elements under such loads is described by both linear and nonlinear elastic properties. Whereas linear elastic moduli of polystyrene are known sufficiently well and their temperature variations are not very large, information on the nonlinear elastic moduli is very limited and their temperature dependence was understudied by now.

In prior investigations employing the acoustoelastic effect, we observed considerable variations in the nonlinear elastic moduli of polystyrene when different nanofillers were added to the polymer matrix [60]. Furthermore, our findings indicated a significant frequency dependence of the nonlinear elastic moduli of pure polystyrene [61], which may be attributed to sub-MHz relaxation processes within the material. In this paper, we applied the developed methodology to analyze the dependencies of nonlinear elastic moduli of polystyrene both on frequency in 0.7–3 MHz range and temperature in 25–65 °C range, which is typical for a wide range of applications. These dependencies are representative of viscoelastic properties of the material and can give information on relaxation processes in it. The data obtained can be used for development of theoretical models and verification/correction of the existing ones.

## 2. Material and Method

Experiments were performed on block samples of commercial styrene copolymer containing 10% wt of ethylene glycol dimethacrylate (EGDMA), produced by Dzerzhinsk Enterprise for Organic Synthesis. Further in the paper we will refer to it as polystyrene (PS). The samples were fabricated in the form of 50 mm long bars, 10 × 10 mm^2^ in cross section.

The methodology applied for measurements of nonlinear elastic moduli of the material was based on the acousto-elastic effect, the details are described in our previous papers [60,61]. Briefly, we analyzed the dependence of the velocities of longitudinal and shear single-frequency ultrasonic waves propagating in the sample on the applied static pressure at different temperatures of the sample. Application of the static pressure of the order of several MPa to the sample caused changes in the time of wave propagation in the sample. The obtained dependencies of velocities of longitudinal and shear waves on the applied pressure allowed for the calculation of the set of nonlinear elastic moduli, as shown below.

**Figure 1:**
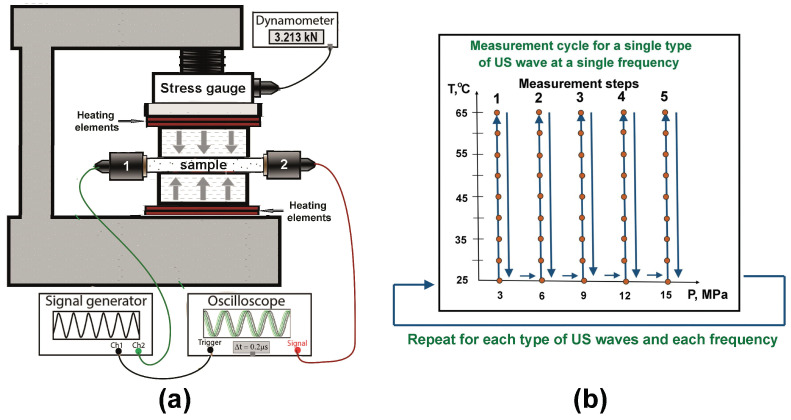
(**a**) Schematics of the experimental setup; (**b**) Flowchart of the measurement procedure. Orange circles indicate individual measurements.

For measurements of variations of the moduli values with temperature, we used the previously developed setup [61] with some minor modifications. The setup comprised a jaw vice, a high-precision stress gauge, a set of piezoelectric transducers used for generation and detection of longitudinal (P121, Amati Acoustics, St. Petersburg, Russia) and shear (V154-RB, Olympus, Waltham, MA, USA) ultrasonic waves, a pulse generator (AM300 Dual Arbitrary Generator, Rohde&Schwarz, Munich, Germany), an oscilloscope (RTB2002, Rohde&Schwarz), and heating elements attached to the jaws. The setup schematics is shown in Figure 1a, the flowchart of the measurement procedure is illustrated in Figure 1b. The pressure applied to the sample was varied within the range of 0–15 MPa and was monitored by the stress gauge. Continuous control was required because temperature variations of the sample could also influence the applied pressure.

The temperature dependencies of nonlinear elastic moduli of polystyrene were analyzed in the temperature range of 25–65 °C. This range guaranteed maintaining the sample in the glassy state even with regard to heating inhomogeneities. The glass transition temperature of polystyrene is about 100 °C, however, softening occurs below this temperature. As shown by Lamberson et al. [62], reproducible results can be obtained in velocity measurements for this material at temperatures below 75 °C.

**Figure 2:**
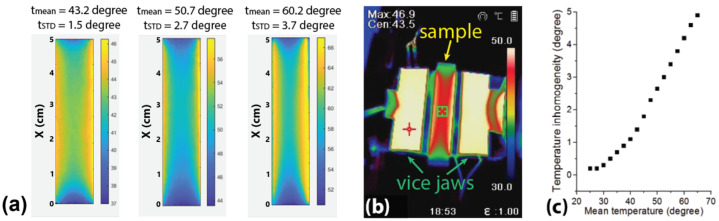
(**a**) Examples of spatial distributions of temperature on the sample surface at different mean temperatures (indicated on top of each image). (**b**) Example of the thermal image of the sample and vise jaws, (**c**) dependence of temperature inhomogeneity in the sample on the mean temperature.

Spatial distributions of temperature in the sample were monitored using a thermal imager B20 (HIKMICRO, Huangzhou, China). The imager was calibrated by comparing its readings with those from thermocouple sensors attached to the sample. So far as the piezoelectric transducers providing generation and detection of ultrasonic waves were applied to the input and output cross-sections of the sample, a completely uniform temperature distribution could not be obtained. Utilization of the thermal imager for analysis of the sample temperature allowed for both determination of the average temperature of the sample in the course of its heating and assessment of temperature heterogeneity over the sample surface due to local heating from the jaws and contact with piezoelectric sensors. The observed temperature heterogeneity rose from 0.2 °C at temperatures close to room to 5 °C at high temperatures of about 65 °C (Figure 2c). Figure 2a presents temperature maps of the polystyrene sample at different mean temperatures, an example of the thermal image of the sample and vise jaws is shown in Figure 2b.

**Figure 3:**
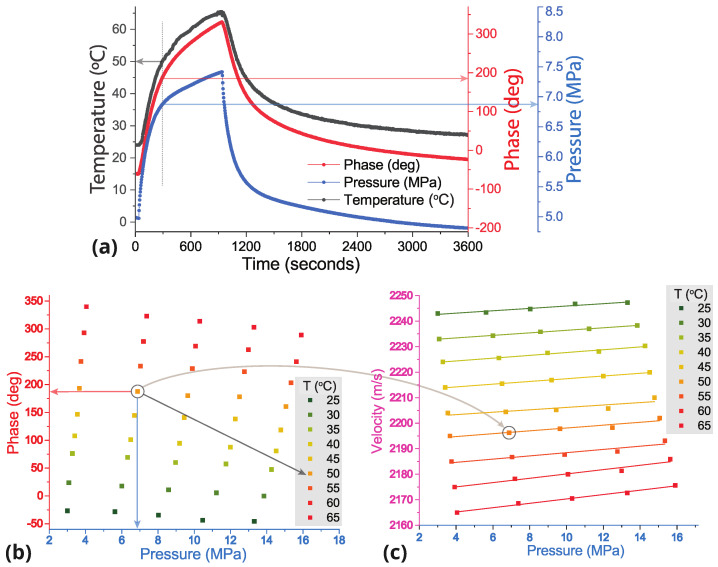
(**a**) Changes in temperature, pressure and phase of the longitudinal ultrasonic wave during one cycle of heating and cooling. The ultrasonic frequency is 1.5 MHz and the initial pressure of the sample at room temperature is 7.5 MPa. (**b**) Phase of the detected ultrasonic wave at different pressures and temperatures of the sample, (**c**) dependence of the velocity of longitudinal ultrasonic waves at 1.5 MHz on the static pressure applied on the sample and sample temperature.

Experiments on the determination of nonlinear elastic moduli at different sample temperatures involved measurements of the velocities of three types of ultrasonic waves (longitudinal and shear with perpendicular directions) at varying pressure and temperature of the sample. Measurements of the phase shift Δϕ of a sinusoidal signal at different temperatures *T* and pressures *P* at a specific frequency of ultrasonic waves *f* allowed us to determine the change in signal transit time as a function of temperature or pressure $\Delta t=\frac{\Delta \phi }{2\pi f}$. Measurements could be carried out either by varying the pressure on the sample at several constant temperatures, or by changing the temperature of the sample at several constant static pressures. Approbation of these experimental scenarios has shown that quite a long time, up to 40–50 min, is required to stabilize the sample temperature (when the system enters a stationary temperature mode) and minimize its fluctuations during the measurement process. At the same time, reliable measurements require high temperature stability during pressure changes, which is why it was taking a significant amount of time to conduct experiments using this scenario. Besides that, an increase in pressure applied to the sample led to a tighter fit of the vice jaws and an increase in the heat transfer rate, which could cause the system to leave the stationary mode and to temperature fluctuations. In this regard, in our experiments the sample was gradually heated from 25 to 65 °C at each static pressure applied to the sample, P≈ [3, 6, 9, 12, 15] MPa. In the course of heating the sample temperature *T*, the phase of the recorded ultrasonic wave ϕ and pressure were monitored. Note that the pressure rose slightly during heating due to sample expansion (see Figure 3a).

The data sets obtained at different static pressures and different temperatures T=25, 30, …, 60, 65 °C provided values of the ultrasonic wave phase and its dependence on the applied pressure. These dependencies were constructed for each temperature: $\Delta \phi (P,T_{1})$, $\Delta \phi (P,T_{2})$, …(see Figure 3b). The velocities of ultrasonic waves as a function of applied pressure were calculated for each specific temperature $T_{i}$ using the equation:

(2) $$V\left(P,T_{i}\right)=\frac{L\cdot V(0,T_{i})}{\Delta t\left(P,T_{i}\right)\cdot V\left(0,T_{i}\right)+L}$$

where $V(0,T_{i})$ is the velocity at zero pressure, the change in the wave propagation time $\Delta t\left(P,T_{i}\right)=\frac{\Delta \phi (P,T_{i})}{2\pi f}$, and *L* is the length of the part of the sample to which pressure was applied. The data obtained for longitudinal ultrasonic waves are plotted in Figure 3c.

It is worth noting that the ultrasonic wave velocity in the sample also depends on temperature. Therefore, it is necessary to apply in Equation (2) the values of $V(0,T_{i})$ for each specific temperature $T_{i}$. The dependence of velocity on pressure at a given temperature does not actually take into account the slight elongation of the sample with increasing pressure, the correct value of the velocity is $V\left(P,T_{i}\right)\left(1+\varepsilon_{x}\right)$, where $\varepsilon_{x}=\nu P/E$ is the sample elongation in *x* direction caused by the applied pressure with ν=λ/(2λ+2μ) being the Poisson’s ratio. The sample elongation due to its compression is taken into account further when using Equations (3)–(5). The procedure used for measuring the dependence of ultrasonic wave velocity on pressure was also described in detail in our recent paper [61].

The obtained dependencies of the ultrasonic wave velocities on pressure allowed calculating the elastic moduli $M_{j}\left(P,T_{i}\right)=\rho \cdot {\left(V_{j}\left(P,T_{i}\right)\right)}^{2}$ for three types of waves: j=x for longitudinal waves, j=y and j=z for shear waves with the displacement vector parallel and perpendicular to the direction of applied pressure, respectively.

Fitting of linear dependencies of the effective moduli $M_{j}\left(P,T_{i}\right)$ obtained at the temperature $T_{i}$ allowed us to determine the dimensionless slope coefficients $\alpha_{x}$, $\alpha_{y}$, $\alpha_{z}$ corresponding to each of the three types of waves. The nonlinear Murnaghan moduli l,m,n were then calculated from the data obtained using the equations [60]:

(3) $$\begin{array}{c} l=-\frac{3\lambda +2\mu }{2}\alpha_{x}-\frac{\lambda (\lambda +\mu )}{\mu }\left(1+2\alpha_{y}\right)+\frac{\lambda^{2}}{2\mu }\left(1-2\alpha_{z}\right), \end{array}$$

(4) $$\begin{array}{c} m=-2\left(\lambda +\mu \right)\left(1+\alpha_{y}\right)+\lambda \left(1-\alpha_{z}\right), \end{array}$$

(5) $$\begin{array}{c} n=-4\mu \left(1+\alpha_{y}-\alpha_{z}\right), \end{array}$$

where λ and μ are linear elastic Lamé moduli measured at zero pressure at each specific temperature, as described in [60]. It is worth noting that at zero pressure the moduli $M_{j}\left(0,T_{i}\right)$ depend only on Lamé moduli λ and μ:

(6) $$\begin{array}{cc} M_{x}\left(0,T_{i}\right) & =\lambda +2\mu , \end{array}$$

(7) $$\begin{array}{cc} M_{y}\left(0,T_{i}\right) & =M_{z}\left(0,T_{i}\right)=\mu . \end{array}$$

## 3. Results and Discussion

### 3.1. Temperature Dependence of Ultrasonic Wave Velocities at Zero Pressure

First, we performed an analysis of temperature and frequency dependencies of the velocity of longitudinal and shear ultrasonic waves in the sample at zero pressure. This data was necessary for further usage in calculating the linear (λ,μ) and nonlinear (l,m,n) elastic moduli using Equations (3)–(5). In experiments, sinusoidal signals with a selected carrier frequency modulated by a Gaussian profile of finite width were generated, and the time of their propagation through the sample was measured at different temperatures in the range of 25–65 °C. Note that too small width (in terms of time) of the Gaussian function led to an increase in the spectral width of the wave packet and its distortion in the course of propagation in the sample; the too large width did not allow the boundaries of the wave packet to be clearly defined due to the smooth increase and decrease in the amplitude of the modulated sinusoidal signal. The typical width (FWHM) of the Gaussian envelope varied from 25 to 35 μs for longitudinal waves of different frequency in the studied range and from 8 to 15 μs for shear waves.

**Figure 4:**
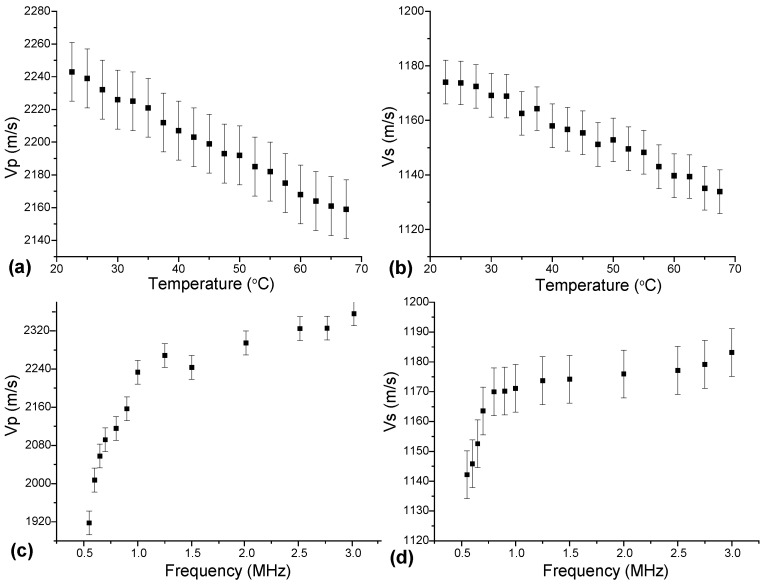
Velocities of longitudinal (Vp) and shear (Vs) ultrasonic waves as a function of temperature at the frequency of 1.5 MHz (**a**,**b**) and as a function of frequency at the temperature of 25 °C (**c**,**d**).

Figure 4 presents the obtained dependencies of velocities of longitudinal and shear ultrasonic waves on temperature at the frequency of 1.5 MHz (Figure 4a,b) and on frequency at the temperature of 25 °C (Figure 4c,d). The data on longitudinal ($V_{p}$) and shear ($V_{s}$) wave velocities measured at different temperatures $V_{i}\left(T\right)$ were used for estimating the effective elastic moduli $M_{j}\left(0,T\right)=\rho V_{i}^{2}\left(T\right)$ and further for calculating the Lamé elastic moduli λ and μ using Equations (6) and (7). The data obtained are summarized in Table 1. As can be seen from Figure 4 and Table 1 both the velocities of longitudinal and shear ultrasonic waves and linear elastic moduli demonstrated a monotonous decrease with temperature. This behavior is in good agreement with the data previously published elsewhere [62,63] and can be explained by rising viscosity of the material at elevating temperatures.

In the given temperature range, the linear elastic moduli decreased linearly with the temperature with slopes dλ/dT=−5.0±0.5 MPa/°C and dμ/dT=−2.2±0.5 MPa/°C, respectively.

### 3.2. Temperature Dependence of Nonlinear Elastic Moduli

The prominent dependence of nonlinear elastic moduli of polystyrene on the frequency of ultrasonic waves has been demonstrated recently in our paper [61], and was explained by the viscoelastic properties of the material. The absolute values of nonlinear elastic moduli of polystyrene were shown to rise considerably with decreasing frequency. For instance, the difference by an order of magnitude has been observed in values of *l* modulus in measurements taken at 3 MHz and 500 kHz. Changes in viscoelastic properties of polystyrene with temperature are supposed to affect the nonlinear elastic moduli as well. These changes were analyzed in this paper at four frequencies of ultrasonic waves of 0.7, 1.0, 1.5 and 3.0 MHz.

**Figure 5:**
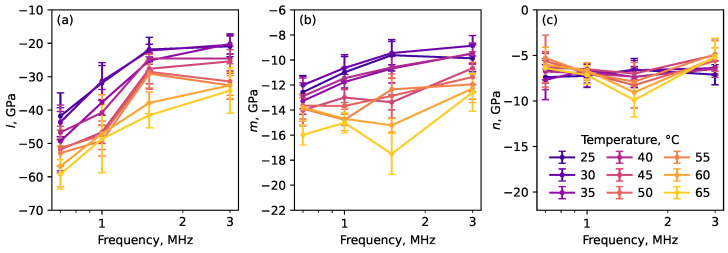
Nonlinear elastic moduli *l* (**a**), *m* (**b**) and *n* (**c**) as a function of frequency at different temperatures. Different temperatures are represented by distinct colors, as shown in panel (**c**).

The determined above values of velocities of longitudinal and shear ultrasonic waves at different temperatures and frequencies and the obtained values of Lamé moduli λ and μ were further used to estimate the nonlinear elastic moduli using the Equations (3)–(5). The results obtained are plotted in Figure 5 and summarized in Table 2 for the lowest (0.7 MHz) and highest (3 MHz) frequencies of the ultrasonic waves used in experiments.

As can be seen from Figure 5 and Table 2, the nonlinear moduli *l* and *m* increased significantly in absolute values with temperature. This temperature dependence is much larger than that for the linear moduli shown in Table 1. At all frequencies in the studied range the increase in temperature from 25 to 65 °C caused increase of the absolute value of the modulus *l* in about 60%, that of modulus *m* in about 20%. At the same time, the modulus *n* was weakly dependent on temperature and frequency and had a high relative measurement error. Indeed, according to Equation (5), the modulus *n* depends on the difference $\alpha_{y}-\alpha_{z}$, which is proportional to a small difference in the sensitivities of the two shear wave velocities to the applied pressure.

**Figure 6:**
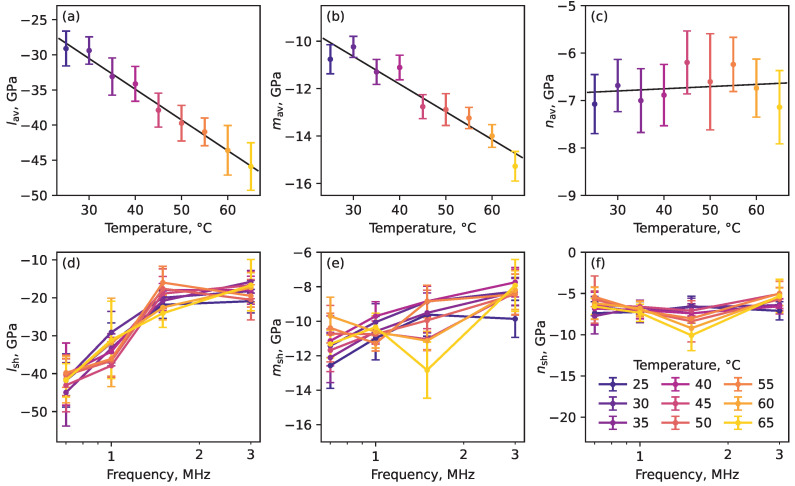
(**a**–**c**) Averaged nonlinear moduli as a function of temperature. (**d**–**f**) Shifted nonlinear moduli as a function of frequency for different temperatures. Different temperatures are represented by distinct colors, as shown in panel (**f**).

We are primarily interested in the overall temperature dependence of the nonlinear moduli. For this purpose, we calculated the nonlinear moduli $l_{\mathrm{av}}$, $m_{\mathrm{av}}$, $n_{\mathrm{av}}$ averaged over the measured frequencies for each given temperature. The resulting dependence of $l_{\mathrm{av}}$, $m_{\mathrm{av}}$, $n_{\mathrm{av}}$ as a function of temperature is shown in Figure 6a–c. One can see the linear dependence on the temperature for $l_{\mathrm{av}}$, $m_{\mathrm{av}}$. For $n_{\mathrm{av}}$, there is almost no dependence on temperature within the measurement tolerance. As a result, we obtained the following slopes $dl_{\mathrm{av}}/dT=-0.44\pm 0.06$ GPa/°C, $dm_{\mathrm{av}}/dT=-0.12\pm 0.04$ GPa/°C, and $dn_{\mathrm{av}}/dT=0.00\pm 0.03$ GPa/°C for the temperature dependence of $l_{\mathrm{av}}$, $m_{\mathrm{av}}$, and $n_{\mathrm{av}}$, respectively. These slopes represent the temperature susceptibility of the nonlinear elastic moduli and are summarized in Table 3.

In general, the nonlinear moduli may have different temperature susceptibilities for each frequency. Therefore, we introduce the shifted nonlinear moduli using the previously obtained frequency-independent slopes:

(8) $$\begin{array}{cc} l_{\mathrm{sh}} & =l-b_{l}\left(T-T_{0}\right), \end{array}$$

(9) $$\begin{array}{cc} m_{\mathrm{sh}} & =m-b_{m}\left(T-T_{0}\right), \end{array}$$

(10) $$\begin{array}{cc} n_{\mathrm{sh}} & =n-b_{n}\left(T-T_{0}\right), \end{array}$$

where $T_{0}=25$ °C is the reference temperature. The obtained values are plotted in Figure 6d–f. One can see a good coincidence of the curves, which means that the temperature susceptibilities of the nonlinear moduli are weakly dependent on the frequency in the studied frequency range.

The nonlinear elastic moduli depend on both frequency and temperature. The frequency dependence has been studied in more detail in our previous work [61]. The observed strong dependence of the nonlinear moduli on frequency was explained by a secondary relaxation process in the sub-MHz range, which is sensitive to static pressure. The molecular origin of this relaxation process remains controversial, but it may be related to the relaxation of some segments or side groups of the polymer chain. Since the relaxation processes have different influence on the sound velocity at different frequencies, one obtains a strong frequency dependence of the nonlinear elastic moduli.

In this paper, we observed a strong dependence of the nonlinear elastic moduli not only on the frequency but also on the temperature. This behavior can be explained by the different influence of pressure on the relaxation processes at different temperatures. The temperature can shift the relaxation time as well as the mechanical coupling with the pressure.

Qualitatively, the dependence of nonlinear moduli on temperature can be explained by the time-temperature superposition principle. At elevated temperatures, all relaxation processes in the polymer are accelerated. This results in a shift of the frequency dependence towards higher frequencies. Since the nonlinear moduli *l* and *m* are negative and increase with frequency, they decrease with temperature. It explains the negative temperature susceptibilities dl/dT and dm/dT. At the same time, the modulus *n* does not depend on frequency, which coincides with almost zero temperature susceptibility dn/dT.

However, the limited frequency range and the measurement errors did not allow us to distinguish the frequency shift from the intrinsic dependence of the nonlinear moduli on temperature. Moreover, the time-temperature superposition principle is reliable only for thermodynamically simple systems, which is rarely the case for systems below the glass transition temperature [53]. Further study is needed to elaborate on this question.

The thermal susceptibility of the nonlinear moduli, with the exception of modulus *n*, is two orders of magnitude larger than the temperature susceptibility of the linear moduli (Table 3). A comparable susceptibility ratio has been observed for vitreous silica [55] and metal-matrix composites [56]. It should be noted that for metal-matrix composites, the modulus *n* similarly exhibited a small dependence on temperature [56].

## 4. Conclusions

We have studied the temperature-frequency dependence of the *nonlinear* elastic moduli of polystyrene using the acousto-elastic effect in the frequency range of 0.7–3 MHz and temperature range of 25–65 °C. At all frequencies in the studied range the increase in temperature caused an increase in the absolute value of the modulus *l* about 60%, that of the modulus *m* about 20%. The modulus *n* was weakly dependent on temperature and frequency and had a high relative measurement error. At the same time the *linear* elastic moduli demonstrated an inverse and relatively weak dependence on temperature in the same range: the moduli λ and μ decreased in 8 and 6%, respectively.

The temperature susceptibility of nonlinear moduli *l* and *m* was two orders of magnitude higher than that of linear moduli λ and μ. For the nonlinear modulus *n*, the temperature variation was less than the measurement error and perhaps of the same order as for λ and μ. The sign of dl/dT and dm/dT as well as the negligible value of dn/dT can be qualitatively explained by the shift of nonlinear moduli towards higher frequencies with increasing temperature. The nonlinear moduli *l* and *m* were strongly frequency dependent in the range of 0.7–3 MHz for all investigated temperatures while the modulus *n* did not depend on frequency. At the same time, the temperature susceptibilities of nonlinear moduli were weakly dependent on frequency within the measurement error.

The reported dependencies are representative of viscoelastic properties of the material and can give information on relaxation processes in them. The data obtained can be useful for development of theoretical models and verification/correction of the existing ones.

## Figures and Tables

**Table 1:** Ultrasonic wave velocities at the frequency of 1.5 MHz and linear elastic moduli λ and μ of the polystyrene sample measured at different temperatures.

Temperature	$V_{p}$ (m/s)	$V_{s}$ (m/s)	ρ (g/cm^3^)	μ (GPa)	λ (GPa)
25 °C	2240 ± 18	1173 ± 8	1.06	1.46 ± 0.01	2.41 ± 0.01
35 °C	2220 ± 18	1162 ± 8	1.06	1.43 ± 0.01	2.36 ± 0.01
45 °C	2200 ± 18	1155 ± 8	1.06	1.41 ± 0.01	2.31 ± 0.01
55 °C	2182 ± 18	1148 ± 8	1.06	1.39 ± 0.01	2.26 ± 0.01
65 °C	2160 ± 18	1135 ± 8	1.06	1.37 ± 0.01	2.21 ± 0.01

**Table 2:** Nonlinear elastic moduli *l*, *m*, *n* of the polystyrene sample measured at different temperatures and indicated frequencies of the ultrasonic wave.

Temperature	*l* (GPa)		*m* (GPa)		*n* (GPa)	
	0.7 MHz	3 MHz	0.7 MHz	3 MHz	0.7 MHz	3 MHz
25 °C	−41.8±7.0	−20.9±3.1	−12.6±1.3	−9.9±1.1	−7.4±1.4	−7.1±1.1
30 °C	−43.6±4.4	−20.4±2.8	−12.0±0.7	−8.9±0.8	−6.5±1.2	−6.4±0.8
35 °C	−49.3±8.9	−20.2±3.0	−13.3±1.5	−9.5±0.8	−7.7±2.2	−6.3±0.7
40 °C	−46.7±8.2	−24.6±3.7	−12.9±1.4	−9.5±0.9	−6.8±2.0	−6.5±0.9
45 °C	−51.8±7.0	−25.4±3.6	−14.0±1.2	−10.7±1.3	−6.3±1.7	−5.0±1.8
50 °C	−51.4±5.4	−31.4±5.3	−13.6±1.6	−11.4±1.2	−5.6±2.9	−5.6±1.4
55 °C	−52.9±3.8	−32.6±3.0	−13.9±0.9	−11.9±1.2	−5.3±1.2	−4.9±1.5
60 °C	−56.8±6.1	−32.6±3.9	−13.8±1.1	−12.3±1.1	−6.0±1.9	−5.2±1.1
65 °C	−59.2±4.4	−34.2±6.8	−16.0±0.8	−12.6±1.5	−6.4±1.0	−5.1±2.0

**Table 3:** Temperature susceptibility of linear (λ and μ) and nonlinear (*l*, *m*, *n*) elastic moduli of the polystyrene sample, averaged over the studied frequency range.

Temperature Susceptibility	Value (GPa/°C)
dλ/dT	−0.0050±0.0005
dμ/dT	−0.0022±0.0005
dl/dT	−0.44±0.06
dm/dT	−0.12±0.04
dn/dT	−0.00±0.03

## Data Availability

Data is available from the authors upon reasonable request.
